# The dynamics of TGF-β in dental pulp, odontoblasts and dentin

**DOI:** 10.1038/s41598-018-22823-7

**Published:** 2018-03-13

**Authors:** Takahiko Niwa, Yasuo Yamakoshi, Hajime Yamazaki, Takeo Karakida, Risako Chiba, Jan C.-C. Hu, Takatoshi Nagano, Ryuji Yamamoto, James P. Simmer, Henry C. Margolis, Kazuhiro Gomi

**Affiliations:** 1 Department of Periodontology, School of Dental Medicine, Tsurumi University, 2-1-3 Tsurumi, Tsurumi-ku, Yokohama 230-8501 Japan; 2 Department of Biochemistry and Molecular Biology, School of Dental Medicine, Tsurumi University, 2-1-3 Tsurumi, Tsurumi-ku, Yokohama 230-8501 Japan; 3 The Forsyth Institute, 245 First Street, Cambridge, MA 02142 USA; 4 Department of Developmental Biology, Harvard School of Dental Medicine, 188 Longwood Avenue, Boston, MA 02115 USA; 5 Department of Biologic and Materials Sciences, School of Dentistry, University of Michigan, 1210 Eisenhower Place, Ann Arbor, MI 48108 USA

## Abstract

Transforming growth factor-beta (TGF-β) is critical for cell proliferation and differentiation in dental pulp. Here, we show the dynamic mechanisms of TGF-β in porcine dental pulp, odontoblasts and dentin. The mRNA of latent *TGF-β1* and *TGF-β3* is predominantly expressed in odontoblasts, whereas the mRNA expression level of latent *TGF-β2* is high in dental pulp. TGF-β1 is a major isoform of TGF-β, and latent TGF-β1, synthesized in dental pulp, is primarily activated by matrix metalloproteinase 11 (MMP11). Activated TGF-β1 enhances the mRNA expression levels of *MMP20* and full-length dentin sialophosphoprotein (*DSPP)* in dental pulp cells, coinciding with the induction of odontoblast differentiation. Latent TGF-β1 synthesized in odontoblasts is primarily activated by MMP2 and MMP20 in both odontoblasts and dentin. The activity level of TGF-β1 was reduced in the dentin of *MMP20* null mice, although the amount of latent TGF-β1 expression did not change between wild-type and *MMP20* null mice. TGF-β1 activity was reduced with the degradation of DSPP-derived proteins that occurs with ageing. We propose that to exert its multiple biological functions, TGF-β1 is involved in a complicated dynamic interaction with matrix metalloproteinases (MMPs) and/or DSPP-derived proteins present in dental pulp, odontoblasts and dentin.

## Introduction

Transforming growth factor-beta (TGF-β) is a signalling molecule that induces cell proliferation, cell differentiation, chemotaxis and apoptosis in monocytes and epithelial, mesenchymal and neuronal cells^1^. Three TGF-β isoforms (TGF-β1, TGF-β2 and TGF-β3) with similar biological activities have been identified in mammals^2,3^. In pulp fibroblasts, synthesis of the collagen matrix is induced by TGF-β1 and TGF-β2 but not by TGF-β3^4^. TGF-β1 also plays a role in tooth development and the reparative process by regulating cell proliferation, differentiation, and reparative dentinogenesis^5,6^. In odontoblasts, TGF-β1 has a crucial role in the transcriptional regulation of two non-collagenous proteins: dentin sialophosphoprotein (DSPP) and dentin matrix protein 1 (DMP1)^7^. TGF-β isoforms have been extracted from the dentin matrices of both rabbit and human teeth^8^, and TGF-β1 has been confirmed to be the predominant isoform, of which approximately half is present in the active form^9^. Our previous findings suggest that DSPP-derived proteins, such as dentin sialoprotein (DSP) and dentin phosphoprotein (DPP) are necessary for maintaining the activity of TGF-β1 in the dentin matrix^10^.

TGF-β is synthesized as a precursor containing a propeptide domain with a TGF-β homodimer^11^ and forms the latent TGF-β complex by interacting with a latency-associated peptide (LAP) after synthesis^12^. Activation of TGF-β is induced by pH^13^, reactive oxygen species^14^, thrombospondin-1^15^ and integrins^16–19^. In addition, proteases such as plasmin and matrix metalloproteinases (MMPs), especially MMP-2 and MMP-9, also activate TGF-β through proteolytic degradation of the latent TGF-β complex^20,21^. Several types of MMPs have been identified in dental pulp, odontoblasts and predentin/dentin^22–27^. The possible roles of TGF-βs in mature odontoblasts are thought to be related to physiological secondary dentin formation, mineralization in intact and healthy teeth, and matrix degradation during dental injury^28^. However, the activation mechanism of TGF-β, the roles of activated TGF-β in dental pulp and odontoblasts, and the inactivation mechanism of TGF-β in dentin matrix are still unclear.

In this study, we performed a series of experiments to understand the dynamic roles and mechanisms of TGF-β in dental pulp, odontoblasts and the dentin matrix. Specifically, we measured the gene expression of TGF-β and its associated MMPs, activation of TGF-β by the MMPs, TGF-β signal induction in odontoblast differentiation, and changes in the amount of DSPP-derived proteins and TGF-β activity with ageing at both the protein and genetic levels.

## Results

### Sample preparation of dental pulp and odontoblasts

**Figure 1:**
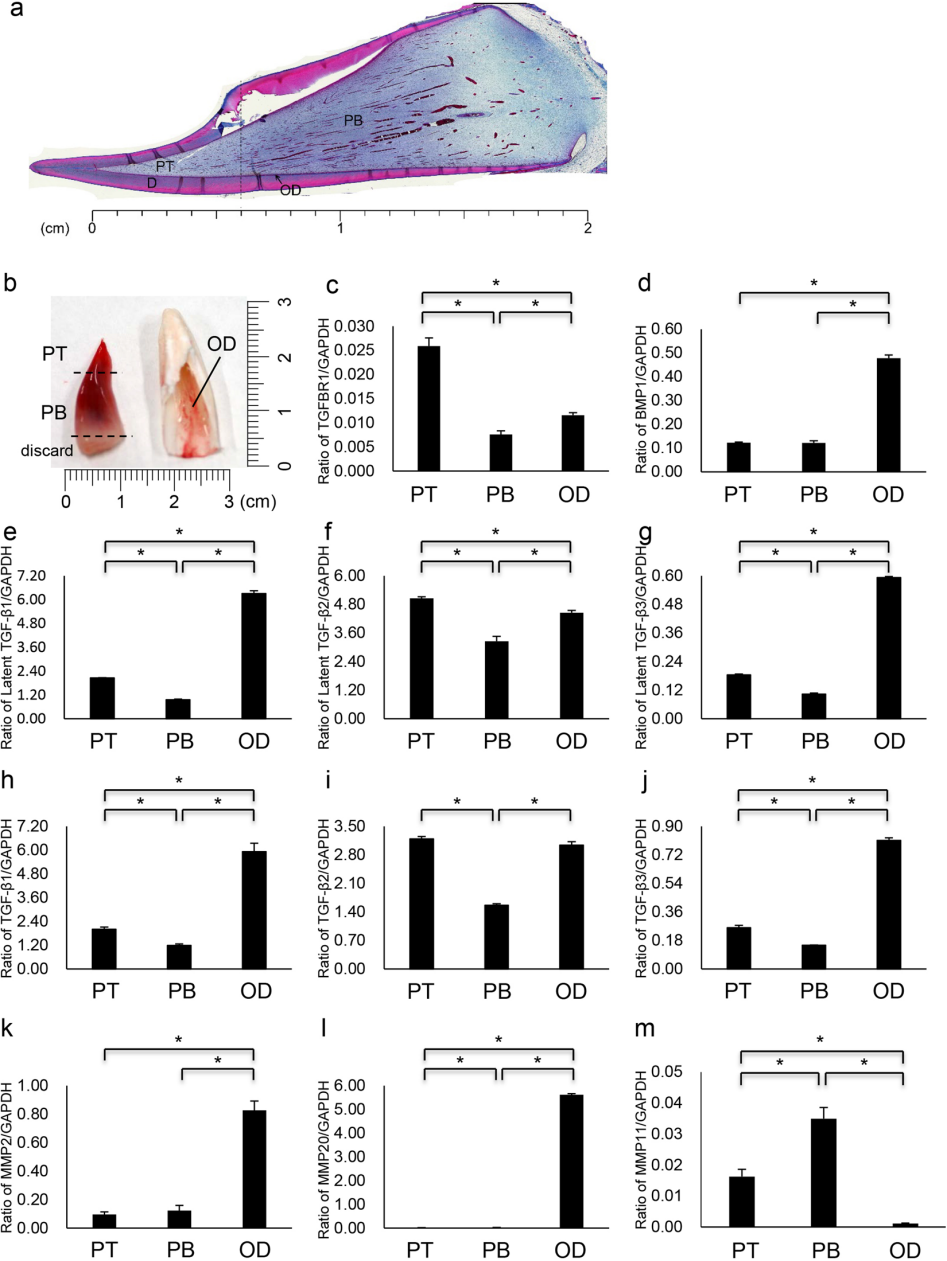
Expression of TGFBR1, BMP1, latent TGF-βs, TGF-βs, and MMPs in porcine dental pulp and odontoblasts. (**a**) Light photomicrograph of tooth germ of 6-month-old porcine permanent incisor by Azan staining. PT: pulp tip, PB: pulp body, OD: odontoblasts and D: dentin. (**b**) Sample preparation for genetic study from permanent incisor and dental pulp from a 6-month-old pig. The pulp tip (PT), pulp body (PB) and bottom portion were excised with a razor blade (left). Following pulp removal, the hollow pulp chamber lined with residual odontoblasts (OD) was used for RNA isolation as the odontoblast sample (right). The mRNA expression of (**c**) TGFBR1, (**d**) BMP1, (**e**) latent TGF-β1, (**f**) latent TGF-β2, (**g**) latent TGF-β3, (**h**) TGF-β1, (**i**) TGF-β2, (**j**) TGF-β3, (**k**) MMP2, (**l**) MMP20 and (**m**) MMP11 was assessed using qPCR. Each expression level was normalized to that of the reference gene glyceraldehyde-3-phosphate dehydrogenase (GAPDH), and the relative quantification data for TGFBR1, latent TGF-βs, TGF-βs, and MMPs in PT, PB and OD were generated on the basis of a mathematical model for relative quantification (n = 6 for each of PT, PB and OD).

We first observed the tooth germ of 6-month-old porcine permanent incisor (Fig. 1a). Azan staining showed the fibrous connective tissue stained blue was localized throughout dental pulp, but the staining intensity of pulp horn area was different from that of pulp chamber area. We designated the area of one-third from the pulp horn as pulp tip (PT), whereas the region of remaining two-thirds as pulp body (PB). We also regarded the residual cells in the hollow pulp chambers after the removal of the pulp as odontoblasts (OD) based on our previous study^29^.

### TGF-β and protease gene expression in porcine pulp and odontoblasts

We investigated TGF-β and protease mRNA expression in porcine dental pulp tissue and odontoblasts. Using qPCR, primer sets that we designed (see Supplementary Tables S1 and S2) and total RNA isolated from PT, PB, and OD (Fig. 1b), we quantified the mRNA expression levels of TGF-β receptor type I (TGFBR1), bone morphogenetic protein 1 (BMP1), inactive TGF-βs (latent TGF-βs), active TGF-βs, and matrix metalloproteases (MMPs). We measured the mRNA expression of latent TGF-βs and active TGF-βs in specific regions to confirm TGF-β mRNA expression. We normalized the TGFBR1, BMP1, latent TGF-β, TGF-β, and MMP expression for the amount of pulp tissue per sample using glyceraldehyde-3-phosphate dehydrogenase (GAPDH) and a standard mathematical model for relative quantification of mRNA expression. TGFBR1 mRNA expression was significantly higher (2.2–3.3-fold) in PT than in PB and OD (Fig. 1c). BMP1 mRNA expression was significantly higher (4.3-fold) in OD than in PT and PB (Fig. 1d). The gene expression of three isoforms of latent TGF-β and TGF-β were detected in PT, PB and OD. Of the three TGF-β isoforms, the mRNA expression levels of latent TGF-β1, TGF-β1, latent TGF-β3 and TGF-β3 were highest in OD: 3.0–6.6-fold for latent TGF-β1 (Fig. 1e), 3.0–5.0-fold for TGF-β1 (Fig. 1h), 3.3–5.9-fold for latent TGF-β3 (Fig. 1g) and 2.0–3.1-fold for TGF-β3 (Fig. 1j). Interestingly, latent TGF-β2 and TGF-β2 mRNA expression levels were highest in PT: 1.1–1.6-fold for latent TGF-β2 (Fig. 1f) and 1.1–2.0-fold for TGF-β2 (Fig. 1i). Among the proteases, the mRNA expression levels of MMP2 (Fig. 1k) and MMP20 (Fig. 1l) were predominantly increased in OD, whereas MMP11 mRNA expression was high in PT and PB, with only trace expression detected in OD (Fig. 1m). MMP11 mRNA expression in PB was significantly higher (approximately 2.1-fold) than that in PT. Although we designed two to six primer sets for MMP1, MMP3, MMP7, MMP8, MMP9, MMP10 and MMP13 (see Supplementary Table S2), none of the amplified products analysed by agarose gel electrophoresis were detectable or of an appropriate size (see Supplementary Fig. S1).

### Immunohistochemical detection of TGF-β1 and TGFBR1 from molar tooth germ

**Figure 2:**
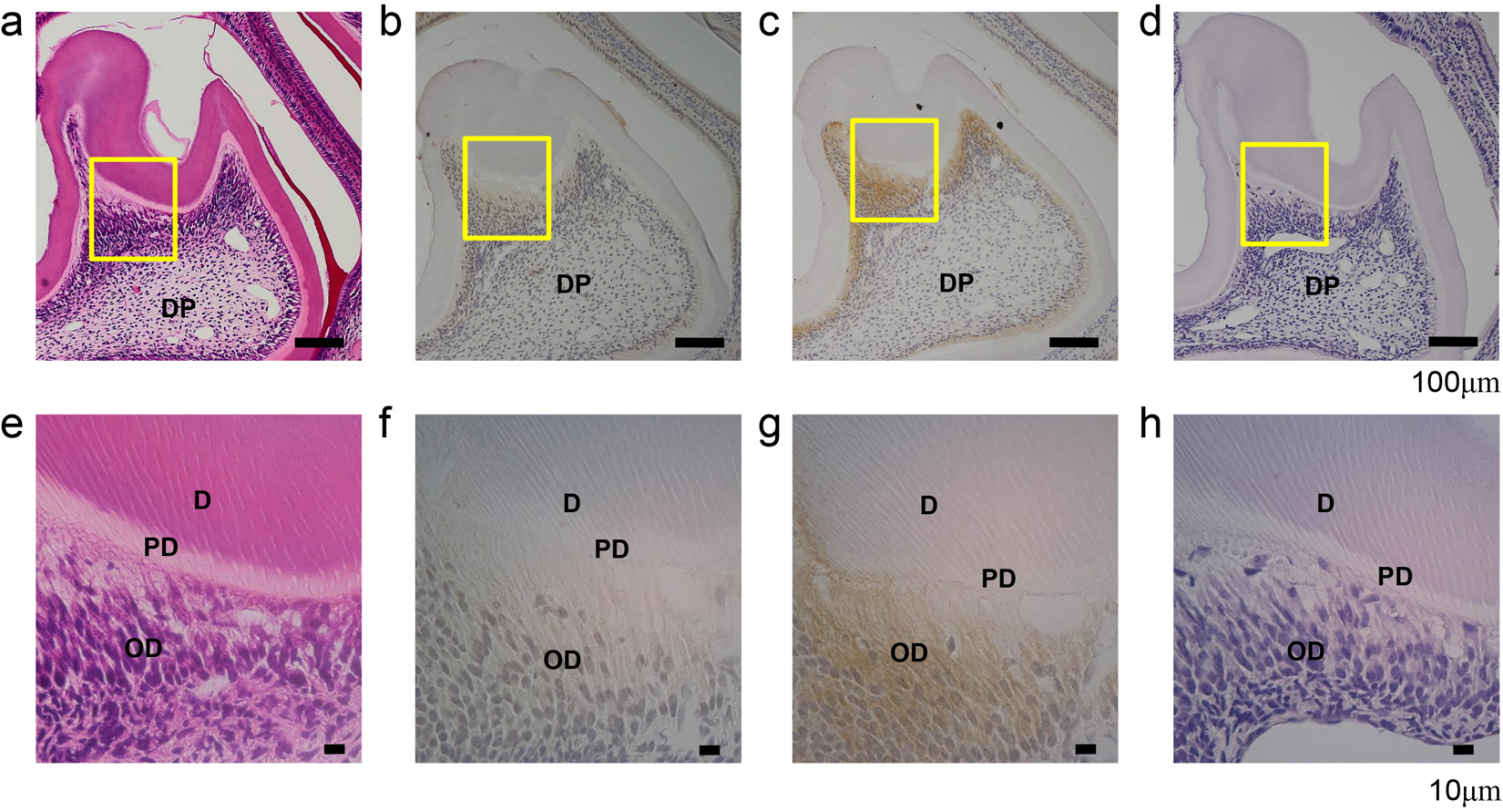
Immunohistochemical detection of TGF-β1 and TGFBR1 from the molar tooth germ of a postnatal day 11 mouse. (**a**,**e**) Haematoxylin-eosin staining, (**b**,**f**) TGF-β1 antibody, (**c**,**g**) TGFBR1 antibody, and (**d**,**h**) absence of primary antibody application as a control. (**a**–**d**) Magnification x100. (**e**–**h**) Magnification x400; the images are a high magnification of the area boxed in (**a**–**d**). D: dentin, OD: odontoblasts, PD: predentin and DP: dental pulp.

Because TGF-β1 and TGFBR1 mRNA were predominantly expressed in porcine odontoblasts, we identified the locations and protein expression levels of TGF-β1 and TGFBR1 in the dentin-pulp complex. Developing mouse first molar tooth bud sections were immunostained using antibodies raised against TGF-β1 and TGFBR1 (Fig. 2). Specific immunostain signals for TGF-β1 (Fig. 2b and f) and TGFBR1 (Fig. 2c and g) were restricted to the layers of odontoblasts and were not observed in dental pulp, predentin or mineralized dentin. Immunostaining for TGF-β1 and TGFBR1 was not detected in the control sections without the primary antibody application (Fig. 2d and h).

### Detection of active forms of TGF-β, MMP2 and MMP11 in dental pulp tissues

**Figure 3:**
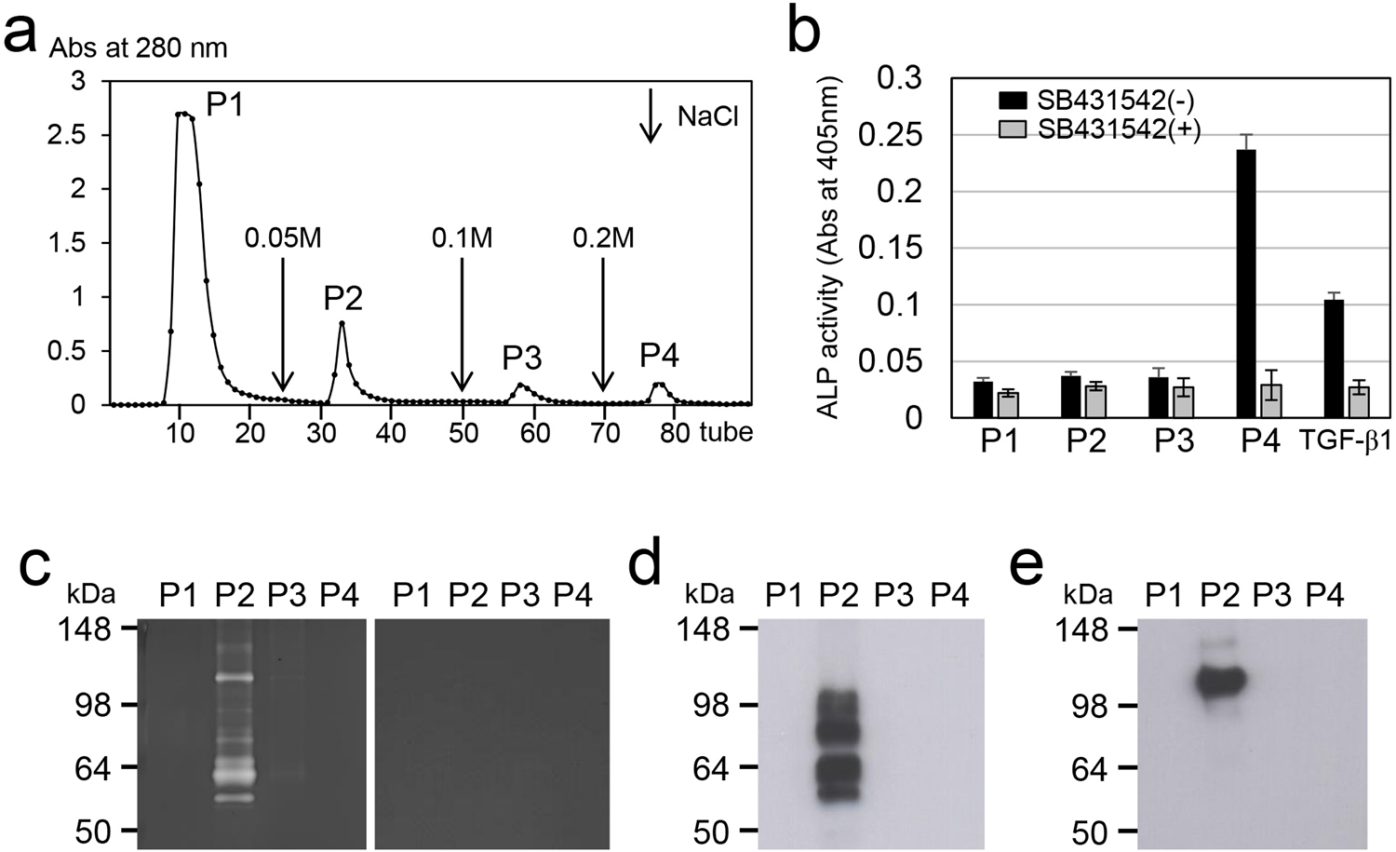
Isolation and detection of active forms of TGF-β, MMP2 and MMP11 in porcine dental pulp. (**a**) Heparin-Sepharose chromatogram of extracts from porcine dental pulp detected at 280 nm absorbance. Downward-pointing arrows represent the starting points of the step gradient with 0.05, 0.1 and 0.2 M NaCl. (**b**) ALP-inducing activity of HPDL cells with (+) or without (−) the addition of SB431542 to fractions P1-P4 isolated by chromatography. Recombinant human TGF-β1 with a carrier (0.3 ng mL^−1^) (TGF-β1) was used as a positive control for the detection of ALP-inducing activity in HPDL cells. The data show the average value of six measurements. (**c**) A gelatin zymogram of fractions P1-P4 incubated without (left) or with (right) EDTA. (**d** and **e**) Western blots of fractions P1-P4 with specific antibodies against (**d**) MMP2 and (**e**) MMP11. The full-length gel (**c**) and blots (**d** and **e**) are presented in Supplementary Fig. S4.

We further investigated the inactive and active forms of TGF-β and proteases in dental pulp. Based on the results of our qPCR analyses, we focused on TGF-β1, MMP2 and MMP11, which were the only molecules detected in dental pulp tissues. We used a Heparin-Sepharose column to separate protein extracts from PB into four fractions (Fig. 3a). Only in the fourth fraction (P4) was enhanced alkaline phosphatase (ALP)-inducing activity in human periodontal ligament (HPDL) cells observed. The activity was significantly reduced by the addition of SB431542, a specific and selective inhibitor of TGF-β type I activin receptor-like kinase receptors (Fig. 3b). Protease activity, as detected by gelatin zymography, was mainly observed in the second fraction (P2) (Fig. 3c, left), and the addition of EDTA completely inhibited this activity (Fig. 3c, right). Western blot analysis of the P2 fraction with an MMP2 antibody revealed multiple bands at approximately 55–100 kDa (Fig. 3d), which were consistent with the observed active bands on the gelatin zymograph. Western blot analysis of the P2 fraction with MMP11 antibody showed a major band at approximately 110 kDa (Fig. 3e) corresponding to an observed active band on the gelatin zymogram.

### *In vitro* activation of TGF-β by MMP2 and MMP11

**Figure 4:**
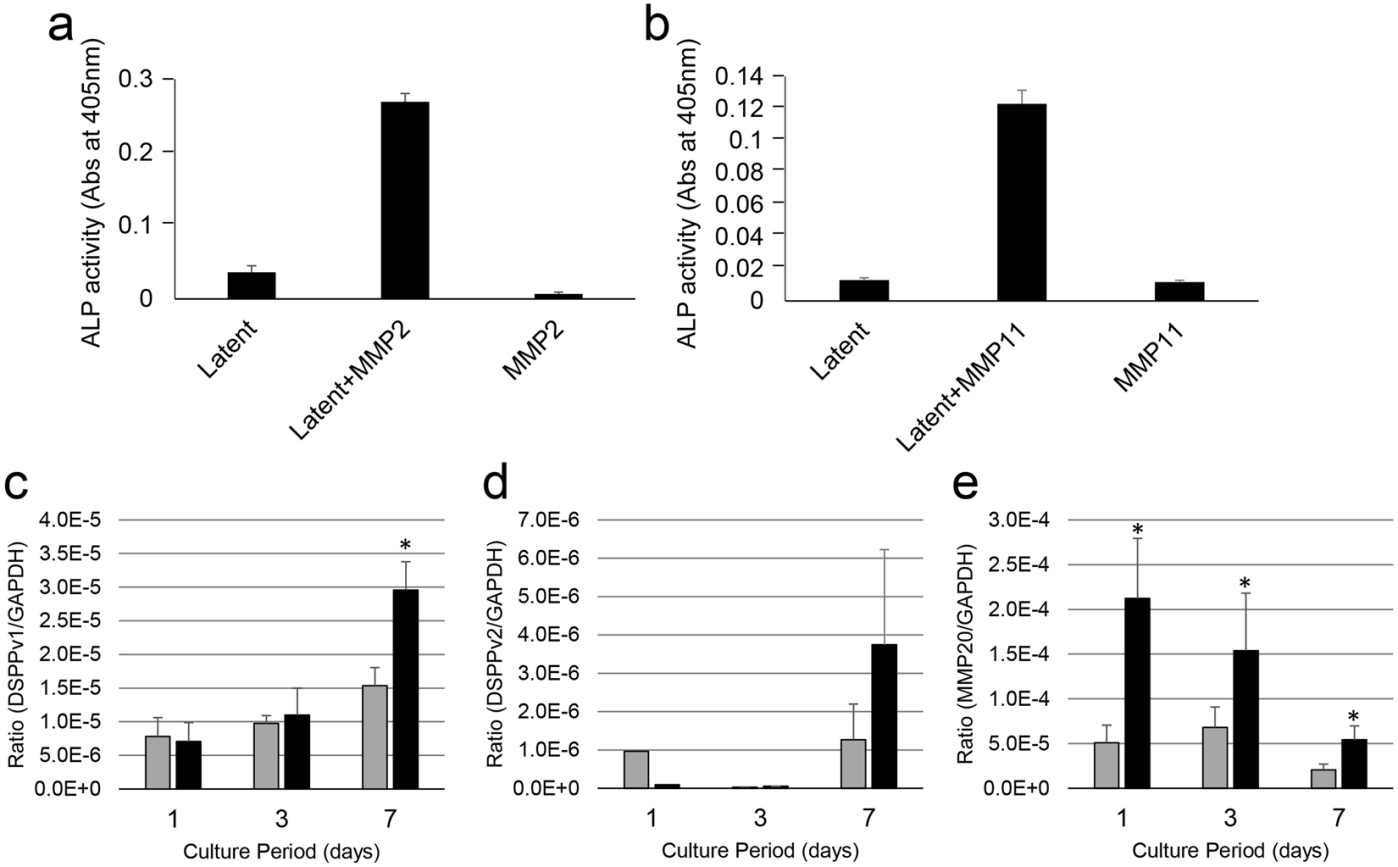
*In vitro* activation of latent TGF-β1 by MMP2 or MMP11 and the effect of activated TGF-β1 on gene expression in the PPU-7 cell line. Latent TGF-β1 was incubated with rhMMP2 or rhMMP11. ALP-inducing activity of HPDL cells exposed to (**a**) latent TGF-β1 only (Latent), latent TGF-β1 with rh-MMP2 (Latent + MMP2) and rh-MMP2 only (MMP2) samples and ALP-inducing activity of HPDL cells exposed to (**b**) latent TGF-β1 only (Latent), latent TGF-β1 with rh-MMP11 (Latent + MMP11) and rh-MMP11 only (MMP11) samples (n = 6). The mRNA expression (as detected by qPCR) of (**c**) DSPP-variant 1 (DSPPv1), (**d**) DSPP-variant 2 (DSPPv2), and (**e**) MMP20. Each mRNA expression value was normalized to that of the reference gene glyceraldehyde-3-phosphate dehydrogenase (GAPDH), and the relative quantification data for DSPPv1, DSPPv2, and MMP20 in PPU-7 were generated on the basis of a mathematical model for relative quantification in a qPCR system (n = 6). Grey bar graph: PPU-7 without TGF-β1, Black bar graph: PPU-7 with TGF-β1.

To understand the activity of MMP2 and MMP11 with TGF-β1 in pulp tissues, we incubated recombinant human latent TGF-β1 (rh-latent TGF-β1) with rhMMP2 (Fig. 4a) or rhMMP11 (Fig. 4b) and determined their ALP-inducing activity in HPDL cells. The incubation of rh-latent TGF-β1 without rhMMP2 or rhMMP11 revealed only trace levels of ALP-inducing activity, whereas treatment with rhMMP2 or rhMMP11 enhanced ALP-inducing activity 9.0-fold and 9.5-fold, respectively, in comparison with the control where no enzyme was present.

### Effect of TGF-β1 on dentin sialophosphoprotein and MMP20 gene expression in dental pulp cells

We further investigated the effect of TGF-β1 on DSPP and MMP20 gene expression using the PPU-7 cell line established from porcine dental pulp cells (see “Immortalization of porcine pulp cells” in the Supplementary Note). Using qPCR, we amplified two mRNA products from the full-length *DSPP* transcript: a segment containing the DGP + DPP coding region (*DSPPv1*) and a smaller segment specific for the DSP-only transcript (*DSPPv2*), and a mRNA product for the MMP20 transcript. The TGF-β1 treatment up-regulated both *DSPPv1* and *DSPPv2* expression in PPU-7 cells at 7 days (Fig. 4c and d). TGF-β1 also dramatically increased *MMP20* expression in just one day, but the *MMP20* level was prone to decrease as time passed (Fig. 4e).

### Status of active TGF-β in *MMP20* null mouse dentin

**Figure 5:**
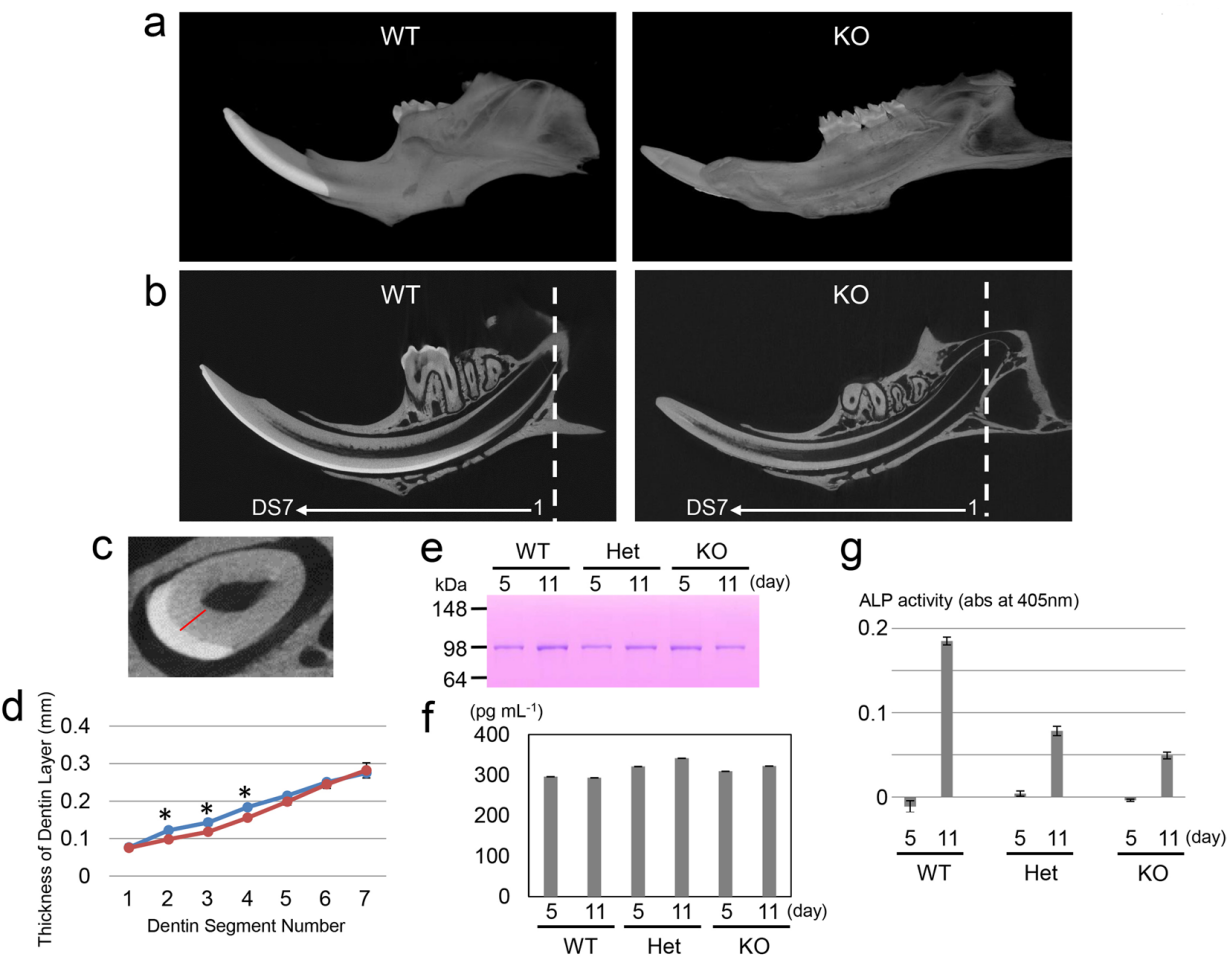
Changes in dentin thickness, dentin phosphoprotein (DPP) and TGF-β activity in the dentin from MMP20 null mice mandibular incisors as measured by µCT. (**a**) Representative three-dimensional reconstructed images of hemi-mandibles of *MMP20*(+/+) and *MMP20*(−/−) mice. (**b**) Representative two-dimensional μCT images of hemi-mandibles cut in the sagittal plane from each mouse genotype. (**c**) Representative two-dimensional cross-sectional μCT images of a mouse mandible incisor at the fourth position (DS4) of seven 1-mm-long cross-sectional dentin segments (DS) starting at the root apex (shown as a dotted line in (b)). The thickness of the dentin layer was measured from the dentin-enamel junction to the dentin surface at the pulp cavity (red line). (**d**) Average dentin thickness measured in seven cross-sectional DS of mouse mandible incisors from each mouse genotype. The line indicates the change in dentin thickness in each dentin segment from *MMP20*(+/+) (blue) and *MMP20*(−/−) (red) mice. The asterisk (*) on the line graph indicates a significant difference between *MMP20*(+/+) and *MMP20*(−/−) samples. (**e**) SDS-PAGE showing the DPP bands from the first molars from *MMP20*(+/+) (WT), *MMP2*(+/−) (Het) and *MMP20*(−/−) (KO) mice at postnatal days 5 and 11. The protein amount was normalized for each mouse genotype on a per-tooth basis. The full-length gel is presented in Supplementary Fig. S4. (**f**) ELISA results for the detection of TGF-β1 in HF extracts obtained from *MMP20*(+/+) (WT), *MMP2*(+/−) (Het) and *MMP20*(−/−) (KO) mice (n = 3) at postnatal days 5 and 11. (**g**) ALP-inducing activity of HPDL cells exposed to HF extracts from *MMP20*(+/+) (WT), *MMP2*(+/−) (Het) and *MMP20*(−/−) (KO) mice (n = 6) at postnatal days 5 and 11.

Based on the qPCR results, we determined that *MMP20* mRNA expression was increased by TGF-β1 in dental pulp cells. A previous study showed that MMP20 activates latent TGF-β1 in the secretory stage of enamel formation^30^, but whether MMP20 activates latent TGF-β1 in dentin is unknown. Therefore, using wild-type (*MMP20*(+/+)), heterozygous (*MMP20*(+/−)) and null (*MMP20*(−/−)) mice and the ALP-HPDL system, we determined *in vivo* TGF-β activity. First, we observed the morphology of the mandibular incisors in 8-week-old *MMP20*(+/+) and *MMP20*(−/−) mice using microcomputed tomography (μCT). Three-dimensional reconstructed images of hemi-mandibles showed that the highly mineralized enamel layer was apparent on the post-eruptive region of the *MMP20*(+/+) mouse incisor but not on the *MMP20*(−/−) mouse incisor (Fig. 5a). Two-dimensional μCT images cut on the sagittal plane showed that the highly mineralized enamel layer was localized in the most mature incisor enamel of the *MMP20*(+/+) mouse incisor but not in the *MMP20*(−/−) mouse incisor (Fig. 5b).

After we confirmed that the enamel layer was different in *MMP20*(+/+) and *MMP20*(−/−) mice, we investigated the dentin layer. We used μCT to measure the relative thickness of the dentin layer in the mouse mandibular incisors of *MMP20*(+/+) and *MMP20*(−/−) mice. We prepared seven 1-mm-long cross-sectional dentin segments (DS) starting at the apex of the root (Fig. 5b and c) and measured the thickness of the dentin layer from the dentin-enamel junction to the dentin surface at the pulp cavity (Fig. 5c). The thickness of the dentin layer increased linearly in both *MMP20*(+/+) and *MMP20*(−/−) mice, but the thicknesses of the DS2, DS3 and DS4 segments in *MMP20*(−/−) mouse incisors were significantly lower (1.24-fold in DS2, 1.21-fold in DS3 and 1.18-fold in DS4) than those observed in *MMP20*(+/+) mouse incisors (Fig. 5d).

Because DS2-DS4 segments are comparable from the secretory stage to the early maturation stage in enamel development^31^, we characterized dentin protein expression and TGF-β1 activity using the first molars from *MMP20*(+/+), *MMP2*(+/−) and *MMP20*(−/−) mice at the secretory (day 5) and early maturation (day 11) stages^32^. We compared the amount of DPP in dentin extracts obtained from the first molars for each genotype using SDS-PAGE (Fig. 5e). We normalized the amount of protein applied per lane to the SDS-PAGE gel for each genotype on a per-tooth basis. The number of teeth used for each group, their collective weights, the total micrograms of protein extracted, and the micrograms of protein extracted per tooth are provided in Supplementary Table S3. DPP was present in 0.17 M HCl/0.98% formic acid (HF) extracts and migrated as a Stains-all-positive single band of approximately 98 kDa on SDS-PAGE. However, there were no significant quantitative changes in the DPP band between each of the genotypes that were assessed (Fig. 5e). An enzyme-linked immunosorbent assay (ELISA) using a TGF-β1 antibody showed that the amount of TGF-β1 in the dentin extracts was nearly equal for each genotype (Fig. 5f). However, the results of ALP-inducing activity assays in HPDL cells on the dentin extracts revealed that the activity of TGF-β1 was significantly different for each genotype. At day 5, each genotype possessed trace levels of ALP-inducing activity in HPDL cells. At day 11, the intensity of ALP-inducing activity in *MMP2*(+/−) and *MMP20*(−/−) mice was 2.7-fold and 3.6-fold lower than that in *MMP20*(+/+) mice (Fig. 5g). We interpret these findings as evidence that MMP20 is required for activation of TGF-β1 in dentin *in vivo*.

### Changes in protein composition and TGF-β activity in dentin

**Figure 6:**
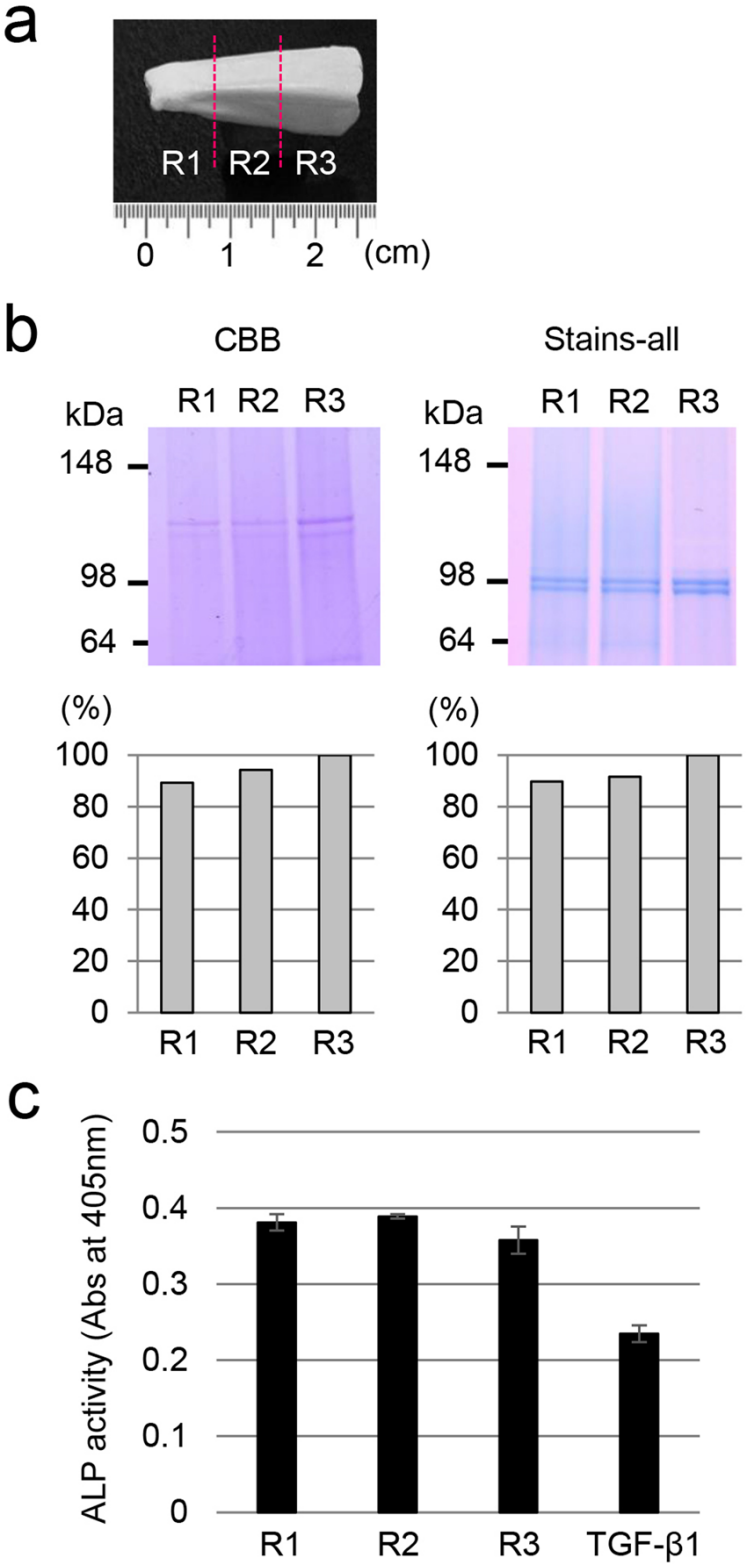
Changes in major dentin proteins and TGF-β activity in porcine incisors. (**a**) A representative image of 6-month-old porcine incisors after the removal of enamel. The incisor was divided into three regions (R1-R3) at 0.8 cm intervals. (**b**) Changes in collagen and DPP in porcine incisor dentin. SDS-PAGE stained with Simply Blue Safe Stain (CBB) for the detection of collagen bands (top, left) and Stains-all for detection of the DPP bands (top, right). The area of the collagen bands (bottom, left) and the DPP bands (bottom, right) on the SDS-PAGE gel were determined using ImageJ software and normalized to the density of R3 in order to compare the protein amounts. The full-length gels are presented in Supplementary Fig. S4. (**c**) ALP-inducing activity of HPDL cells exposed to each of the G2 extracts from R1-R3. The data show the average value of six measurements. TGF-β1: recombinant human TGF-β1 with a carrier (0.3 ng mL^−1^).

Because there are different levels of TGF-β activity in immature and mature enamel from 6-month-old porcine second molars^30^, we determined TGF-β activity in the dentin of 6-month-old porcine incisors by measuring ALP-inducing activity in HPDL cells. Following enamel removal, we cut the incisors into three regions (R1-R3) at 0.8 centimetre intervals (Fig. 6a) and crushed and ground them to prepare dentin powder samples. The dentin powder of each region was decalcified with HCl-formic acid, extracted with Tris-guanidine (G2 extract) buffer, and analysed by SDS-PAGE stained with Simply Blue Safe Stain (CBB) (Fig. 6b, top, left) and Stains-all (Fig. 6b, top, right). The amounts of G2 extract obtained from the dentin powder of the R1 (2.60 g), R2 (2.37 g) and R3 (1.57 g) regions were 29.5 mg, 44.3 mg and 36.2 mg, respectively. We normalized each protein sample amount for SDS-PAGE by calculating the amount of G2 extract per gram of teeth that was loaded onto the SDS-PAGE gel. Collagen and Stains-all-positive DPP doublet bands from the G2 extracts had molecular weights of 120 kDa and 98 kDa, respectively. The intensity of the collagen (Fig. 6b, bottom left) and DPP (Fig. 6b, bottom right) bands from the R1 and R2 regions were approximately 90% less than those from the R3 region. We also determined the ALP-inducing activity in HPDL cells enhanced by TGF-β contained in each of the G2 extracts obtained from the R1-R3 regions. The TGF-β activity remained at approximately the same level for all three regions (Fig. 6c).

### Changes in the composition of porcine root dentin over time

**Figure 7:**
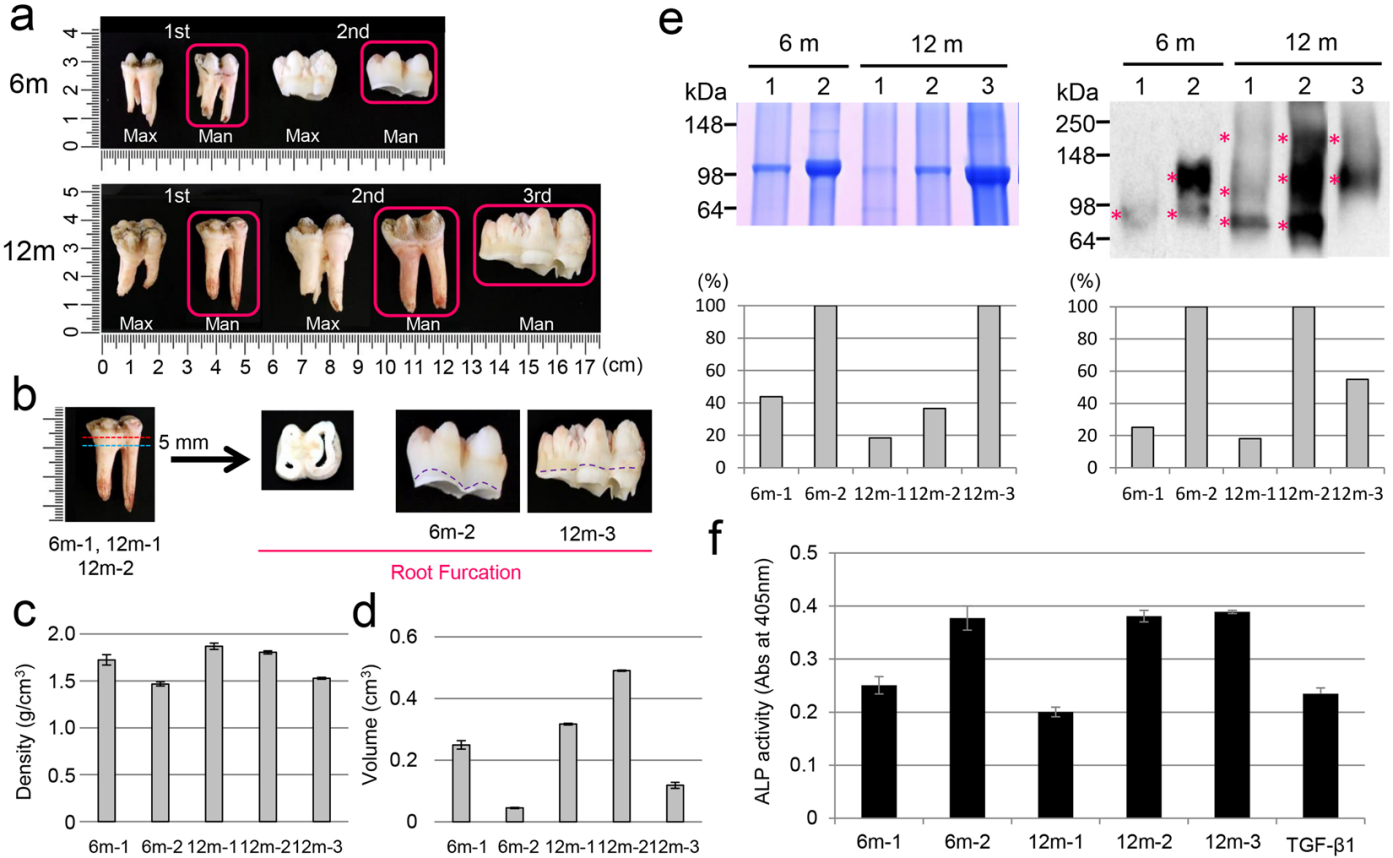
Changes in dentin composition over time. (**a**) Images of maxillary and mandibular first, second and third molars of 6- and 12-month-old pigs. 6 m: 6-month-old pig, 12 m: 12-month-old pig, 1st: first molar, 2nd: second molar, 3rd: third molar, Max: maxillary, Man: mandibular. Mandibular first (6^m−1^, 12^m−1^), second (6^m−2^, 12^m−2^) and third (12^m−3^) molars, as indicated by a red square, were used for this study. (**b**) Schematic representation of the root furcation preparation. The apical roots of 6^m−1^, 12^m−1^ and 12^m−2^ teeth were first separated by cutting with a jewellery saw (blue dotted line), and then, each of the root furcations (approximately 5 mm thick) were prepared by cutting at the cementum-enamel junction (CEJ) (red dotted line). The root furcations from 6^m−2^ and 12^m−3^ teeth were obtained by cutting at the CEJ with dissecting scissors (purple dotted line). (**c**) The densities of the porcine molars were measured using a pycnometer (n = 3). (**d**) Volumes of the porcine molars were calculated from the measured densities (n = 3). (**e**) Changes in the amount of DPP and DSP in the porcine molars. SDS-PAGE stained with Stains-all for the detection of DPP bands (top left) and western blots using a specific antibody against DSP (top right), showing AN extracts from each root furcation. Each protein sample amount for SDS-PAGE was normalized (see Supplementary Table S2). The areas of the DPP bands on the SDS-PAGE gel (bottom, left) and the DSP bands (bottom, right) on the western blots were determined using ImageJ software and normalized to the density of the 6^m−2^ and 12^m−3^ samples in order to compare the protein amount at each age. The areas of the DSP bands at each age were calculated as the total area of the band in each lane shown with asterisks. The full-length gel and blot are presented in Supplementary Fig. S4. (**f**) ALP-inducing activity of HPDL cells exposed to each of the AN extracts (n = 6). TGF-β1: recombinant human TGF-β1 with a carrier (0.3 ng mL^−1^).

Since TGF-β1 activity in dentin is retained by binding to DPP and DSP^10^, we assessed whether the DSPP-derived proteins, specifically, DPP and DSP, changed over time. We used non-carious 6-month-old first (6^m−1^) and second (6^m−2^) molars and 12-month-old first (12^m−1^), second (12^m−2^) and third (12^m−3^) molars (Fig. 7a) and prepared each of the root furcations for this experiment (Fig. 7b). We measured the density of the root furcation at each age using a pycnometer and calculated both density and volume (see Supplementary Table S4). The dentin density of the teeth, ordered from high to low, was 12^m−1^, 12^m−2^, 6^m−1^, 12^m−3^ and 6^m−2^ (Fig. 7c), whereas the volume ordered from high to low was 12^m−2^, 12^m−1^, 6^m−1^, 12^m−3^ and 6^m−2^ (Fig. 7d). We also normalized the amount of protein in each sample for SDS-PAGE by calculating the amount of total protein in the 0.5 M acetic acid/2 M NaCl (AN) extract per volume of each root furcation (see Supplementary Table S5 and “Density and volume measurement” in Supplementary Note) and loaded that amount onto the SDS-PAGE gel. The samples in order of the highest to lowest amount of DPP were 6^m−2^ = 12^m−3^, 6^m−1^, 12^m−2^ and 12^m−1^ (Fig. 7e, top, left). The level of the DPP band for 6^m−1^ dropped to approximately 40% of that of 6^m−2^ (Fig. 7e, bottom, left). The level of the DPP bands in 12^m−1^ and 12^m−2^ molars decreased to approximately 20 and 40% that of 12^m−3^ molars, respectively (Fig. 7e, bottom left). Western blotting showed that the molecular weight of the DSP band was prone to be reduced with age (Fig. 7e, top right). DSP expression in the 6^m−1^ and 12^m−1^ samples was approximately 20% that of the 6^m−2^ and 12^m−2^ samples, respectively (Fig. 7e, bottom right). The expression of DSP in the 12^m−3^ sample was approximately 60% that of the 12^m−2^ sample (Fig. 7e, bottom right). The proportion of ALP-inducing activity in HPDL cells from 6^m−1^ and 12^m−1^ teeth was approximately 50% that of the 6^m−2^, 12^m−2^ and 12^m−3^ samples (Fig. 7f).

## Discussion

During tooth formation, in the dental pulp, the stage of odontoblast near the pulp horn is more advanced than that of the odontoblast in the pulp chamber. Our previous study has shown that the mRNA level of DSPP variant was different between pulp horn and pulp chamber area^33^. This finding led us to believe that both area might show a different gene expression. In the present study, we demonstrated that the dental pulp of 6-month-old porcine permanent incisor was stained blue by Azan staining, which is used for the detection of connective tissue. Its intensity, however, was different between pulp horn and pulp chamber area. Based on these information, we considered the region of one-third from the pulp horn and the region of remaining two-thirds as pulp tip (PT) and pulp body (PB), respectively. At the periphery of the dental pulp, odontoblast cell bodies form a layer infiltrated by capillaries. Our previous study using light microscopy has been shown that the retention of odontoblasts in pig teeth are observed on the surface of predentin following the removal of the dental pulp^29^. Based on this information, we regarded the residual cells in the hollow pulp chambers after the removal of the pulp as odontoblasts.

TGF-β1 is the predominant isoform detected in human dentin and the major isoform in rabbit dentin, where small amounts of TGF-β2 and TGF-β3 isoforms are detected^8^. We showed that the mRNA expression levels of TGFBR1, BMP1, TGF-β1, TGF-β3, latent TGF-β1 and latent TGF-β3 were predominant in odontoblasts, while those of TGF-β2 and latent TGF-β2 were present in both dental pulp and odontoblasts. Our findings suggest that a certain amount of TGF-β2 is also present in porcine dentin matrix, although TGF-β1 is the main isoform.

MMP mRNA is expressed in pulp and odontoblasts, where MMPs play an important role in dentin matrix formation. MMP2, MMP3, MMP8, MMP9, MMP14 and MMP20 are the main MMPs that have been identified in pulp, odontoblasts and predentine/dentin^22–27^. MMP13 has been found in radicular dentin. We have demonstrated in pigs that the mRNA expression levels of MMP2 and MMP20 are predominant in odontoblasts. In addition, we discovered that MMP11 mRNA expression is predominant in dental pulp and revealed that MMP11 mRNA expression was different in the tip and the body. These findings suggest that analysis of mRNA levels of those three MMPs may be useful for assessing the differentiation of odontoblasts.

Both TGF-β1 and TGFBR1 were detected in the inner dental epithelium of the first molar in mouse embryos at E18.5, and their expression levels were elevated in secretory ameloblasts and odontoblasts in postnatal day 3 mice^34^. Immunohistochemical analysis in developing murine teeth has shown that TGFBR1 expression occurs at the apical ends of secretory-stage ameloblasts interfaced with the enamel matrix^34^. Our previous report showed that TGFBR1 in secretory-stage ameloblasts is necessary for TGF-β1 autocrine regulation during enamel formation^30^. In dentin, both TGFBR1 and TGFBR2 are localized in odontoblasts (as detected by immunohistochemistry)^9^. TGF-β signalling controls odontoblast maturation and dentin formation during tooth morphogenesis^35^. The odontoblasts of developing mouse teeth in postnatal day 11 mice were immunostained with TGF-β1 and TGFBR1 antibodies. From these collective findings, the presence of TGF-β1 and TGFBR1 in secreting odontoblasts in this study suggests that TGF-β signalling may play a key role in synthesis of dentin proteins and/or proteases and may provide the basis for an increasing autocrine effect of local TGF-β1 on odontoblast development as well as enamel formation. Our data further suggest that TGFBR1 in odontoblasts may have been prepared at any time to be able to respond to TGF-β1, even after the completion of dentin formation.

Both mature TGF-β1 and TGF-β2 possess one heparin-binding site, while mature TGF-β3 does not (see Supplementary Fig. S2 and “Extraction of MMPs and TGF-β from porcine dental pulp” in Supplementary Note). In addition, both MMP2 and MMP11 also have TGF-β binding sites in the catalytic domain (see Supplementary Fig. S3 and “Extraction of MMPs and TGF-β from porcine dental pulp” in Supplementary Note). Based upon this information, we isolated protein from the dental pulp with heparin affinity chromatography and were able to detect active forms of MMP2 and MMP11 in the P2 fraction eluted with 50 mM NaCl, and TGF-β activity in the P4 fraction eluted with 200 mM NaCl. This finding suggests that TGF-β possesses a high heparin binding affinity than MMPs.

TGF-β is generally activated by pH^13^, reactive oxygen species^14^, thrombospondin-1^15^ and integrins^16,18,19^. In addition to those factors, proteases such as plasmin, MMP2 and MMP9 are also able to activate TGF-β through proteolytic degradation of the latent TGF-β complex^20,21^. We demonstrated that rh-latent TGF-β1 was dramatically activated by MMP2 and MMP11 *in vitro*. Combined with the mRNA expression of MMPs, our data suggest that *in vivo* activation of TGF-β in dental pulp is caused by both MMP2 and MMP11.

Three mammalian isoforms of TGF-β, namely, TGF-β1, TGF-β2 and TGF-β3, are potent regulators of cell growth, cell differentiation and extracellular matrix deposition^36^. *In vitro* TGF-β1 and/or TGF-β3 are able to stimulate matrix secretion in odontoblasts, are mitogenic to pulp cells, and possess a potential inductive effect for cell differentiation of dental pulp cells^37^. We previously generated two PCR amplification products from the full-length DSPP (DSPPv1) and the DSP-only (DSPPv2) transcripts and found that both DSPPv1 and DSPPv2 products are predominantly observed in odontoblasts, while only trace expression of the DSPPv1 transcript is detected in dental pulp^33^. We also demonstrated that the mRNA expression level of DSPPv1 is significantly enhanced by TGF-β1 in the PPU-7 cell line over a 7 day experimental period, whereas that of DSPPv2 was only slightly increased. These findings suggest that TGF-β1 is one of the regulators of odontoblast differentiation and may be a useful differentiation marker for odontoblasts.

TGF-β1 has been reported to induce modest down-regulation of MMP20 in mature human odontoblasts^38^. We demonstrated *in vitro* that TGF-β1 up-regulates the MMP20 mRNA expression level in dental pulp cells in a day, but the level is decreased over time. Since TGF-β1 and MMP20 mRNA expression is high in odontoblasts, this finding suggests that the up-regulation of MMP20 mRNA by TGF-β1 may constantly occur in odontoblasts *in vivo*. Our data also suggest that the mechanisms by which TGF-β1 regulates MMP20 mRNA expression may be different between dental pulp cells and mature odontoblasts.

Backscatter scanning electron microscopy images from 9-week-old mouse incisors shows that the dentin of both wild-type and *MMP20* null mice exhibit nearly equal levels of mineralization^39^, but the dentin densities are different between wild-type and *MMP20* null mice^40^. We demonstrated that the thickness of the dentin layer indicated significant differences in the DS2, DS3 and DS4 segments from *MMP20*(+/+) and *MMP20*(−/−) mice, but the amount of DPP in dentin was not significantly different between *MMP20*(+/+)*, MMP2*(+/−) and *MMP20*(−/−) mouse molars taken at postnatal days 5 and 11. However, the present study demonstrated that the TGF-β1 activity level was drastically reduced in proportion to the reduction in MMP20 between the dentin extracts of all three genotypes of 11-day-old mice (*i.e., MMP20*(+/+) > *MMP20*(+/−) > *MMP20*(−/−)), although the amount of latent TGF-β1 produced in dentin was nearly equal among all three mouse genotypes. Given that the ALP-inducing activity in *MMP20*(−/−) mice was drastically reduced (3.6 times lower than that of *MMP20*(+/+) dentin), we concluded that *in vivo* activation of TGF-β1 in dentin was primarily induced by MMP20.

Transgenic mice overexpressed TGF-β1 in odontoblasts using the *DSPP* gene promoter develop distinct dentin defects similar to those seen in human dentin dysplasia and dentinogenesis imperfecta^41^ Moreover, TGF-β1 knockout mice dentition shows the widespread pulp and less mineralized dentin^42^. We found that the thickness of the dentin layer was significant low in the DS2, DS3 and DS4 segments in *MMP20*(−/−) mice as described above. This finding suggests that the loss of TGF-β1 activity may affect the mineralization of the dentin layer.

In developing porcine enamel, the autocrine regulation of TGF-β1 through MMP20 coincides with other reactions related to MMP20 in secretory-stage enamel, and TGF-β1 activity is maintained by binding a water-soluble amelogenin and induces TGF-β signalling through TGFBR1 on ameloblasts^30^. Upon degradation of the enamel matrix components during the maturation stage, TGF-β1 activity decreases^30^. In the case of dentin, dentin non-collagenous proteins, especially proteoglycans, are important for sequestration of TGF-β1 in the dentin matrix^43^. Porcine DSPP is the most abundant non-collagenous protein in dentin. DSPP is processed by proteases into 3 independent proteins: DSP^44^, dentin glycoprotein (DGP)^45^, and DPP^46^. Of these, DSP is a highly glycosylated proteoglycan containing both chondroitin 4- and chondroitin 6-sulfate chains^44,47^. We have previously reported that TGF-β1 activity in porcine dentin is rescued by binding not only to DSP but also to DPP^10^. This study demonstrated that the proportion of collagen, DPP and TGF-β1 was almost unchanged in all regions of dentin in the incisors from a 6-month-old pig. This finding suggests that TGF-β1 has different roles in enamel and dentin. Considering that the loss of MMP20 affected the thickness of dentin in some parts of the tooth, our data also suggest that MMP20 regulates not only TGF-β1 activation but also the processing and/or degradation of dentin proteins.

Dentin is made up of approximately 70% inorganic materials, 20% organic materials and 10% water. Due to the continual deposition of peritubular dentin, primary dentin gradually shows a reduced change in the diameter of the dentinal tubules with age. Even after tooth eruption, physiological secondary dentin is formed in the pulp cavity. Because secondary dentin is deposited on the entire pulpal surface of the dentin, the pulp horns are obliterated, and the pulp cavity eventually becomes smaller. In addition, the density determinations were made on sound dentin from human teeth of various ages^48^. In the present study, we tried a new method to determine the change in the organic components of dentin in porcine root furcations with age, especially DSPP-derived proteins and TGF-β. We demonstrated that both dentin density and volume were higher as teeth grew, and DPP and DSP were prone to degradation with age. These findings suggest that the dentin composition, especially DSPP-derived proteins, changes with age and that DSPP-derived proteins and TGF-β are transiently expressed in root dentin.

**Figure 8:**
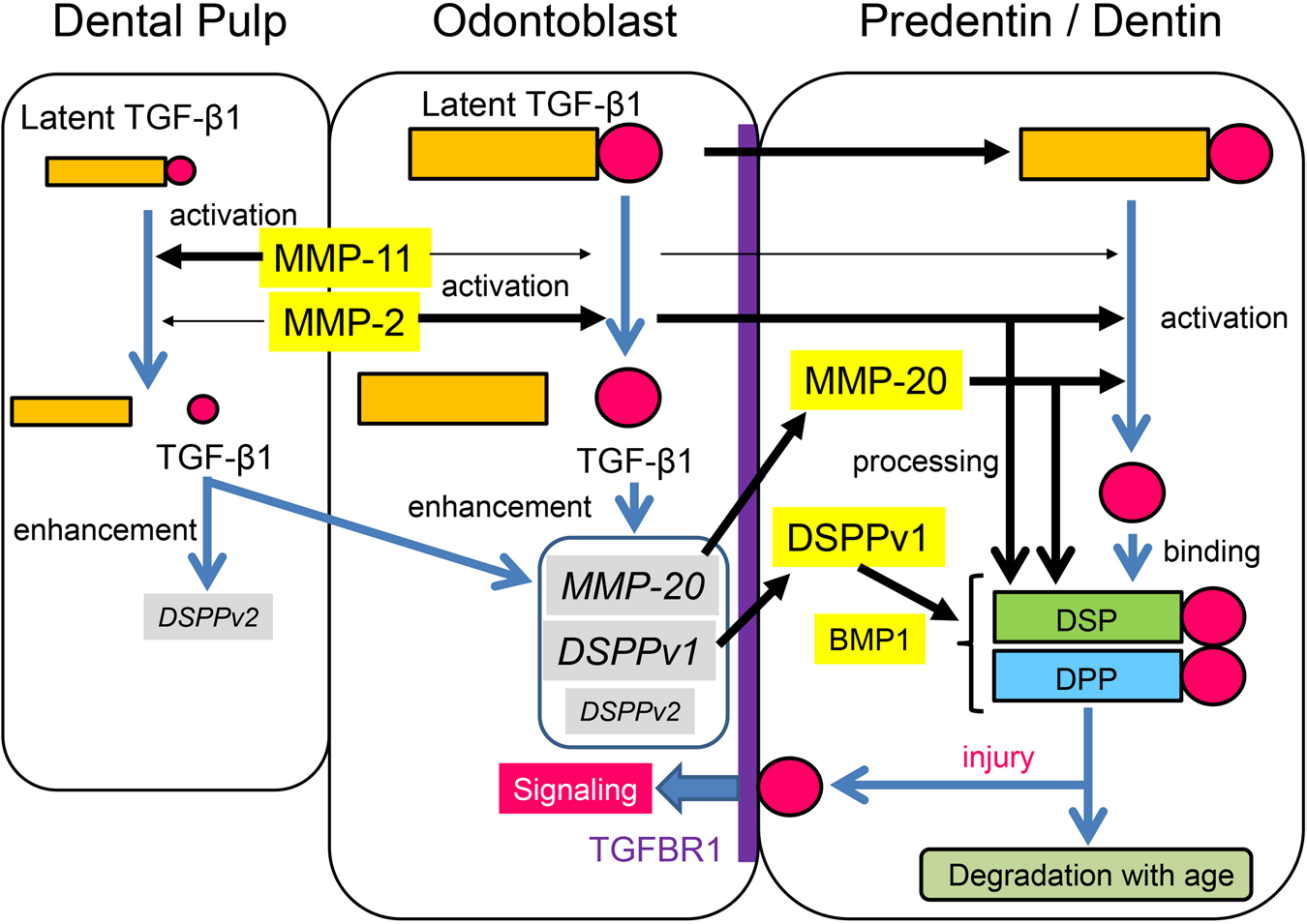
Proposed dynamic mechanism of TGF-β1 in porcine dental pulp, odontoblasts and dentin.

We propose a dynamic mechanism for TGF-β, mainly TGF-β1, in dental pulp, odontoblasts and dentin (Fig. 8). Latent TGF-β1 synthesized in dental pulp is primarily activated by MMP11 and to a lesser extent by MMP2. In contrast, latent TGF-β1 synthesized in odontoblasts is mainly activated by MMP2. Activated TGF-β1 enhances the mRNA expression levels of MMP20 and full-length DSPP, coinciding with the promotion of odontoblast differentiation. Latent TGF-β1 synthesized in odontoblasts is also secreted in dentin matrix and primarily activated by MMP2 and MMP20 and to a lesser extent by MMP11. Previous studies suggest that the active form of TGF-β1 binds to DPP and DSP^10^, which are processed from DSPP by BMP1, MMP2 and MMP20^49,50^, to maintain its activity and is present in the dentin matrix rather than actively signalling. Although DPP-TGF-β1 and DSP-TGF-β1 complexes decrease with age, they provide a reservoir of bioactive molecules that influence cell behaviour in the dentin-pulp complex following tissue injury, such as carious invasion. Based on the fundamental knowledge obtained in this study, further studies are required to elucidate how the degradation process of DPP and DSP changes over time and the molecular mechanisms underlying the formation of reparative dentin.

## Methods

All animal experiments, except for “Changes in dentin composition over time”, were approved by the Institutional Animal Care Committee and the Recombination DNA Experiment and Biosafety Committee of the Tsurumi University School of Dental Medicine. The “Changes in dentin composition over time” experiment was approved by the Institutional Animal Care and Use Program at the University of Michigan.

Mouse experiments using *MMP20* null and wild-type littermates were performed in accordance with protocols approved by The Forsyth Institute’s Institutional Animal Care and Use Committee.

### Preparation of pig samples

For experiments with porcine teeth, tooth germs of permanent incisors were surgically extracted from the mandible of deceased 6-month-old pigs from the Meat Market of the Metropolitan Central Wholesale Market (Shinagawa, Tokyo, Japan) and used for gene (n = 6) and protein (n = 10) studies, and permanent molars (n = 10) were used to detect the presence of MMPs in dental pulp. Moreover, tooth germs of permanent molars were surgically extracted from the mandibles of deceased 6- and 12-month-old pigs from the Dunbar Meat Packing Co. (Milan, MI, USA) and used for density and volume measurements and protein studies (n = 3).

### Preparation of tooth germ cells

Dental pulp samples were removed from the porcine incisors described above, and their surfaces were carefully cleaned with Kimwipes (Kimberly Clark Corp. Irving, TX, USA) to avoid contamination of the odontoblasts. Then, the samples were separated into tip and body segments using a razor blade^33^ (Fig. 1a). The pulp samples were either used immediately for RNA isolation or stored at −80 °C until needed for protein extraction. Following the removal of the pulp, the residual odontoblasts in the hollow pulp chambers were used for RNA isolation.

### Quantitative real-time PCR

Quantitative real-time PCR (qPCR) of the total pulp RNA was performed using the SYBR Green technique on a LightCycler Nano system (Roche Diagnostics, Mannheim, Germany) (see “Quantitative real-time PCR” in Supplementary Methods). The specific primer sets and reaction conditions are shown in Supplementary Tables S1 and S2.

### Histological study

Formalin-fixed paraffin-embedded murine mandibles or porcine tooth germ were sectioned. For murine mandibles, the immunohistochemical analysis was performed using anti-TGF-β1 (#orb7087, Biorbyt, Cambridge, UK) and anti-TGFBR1 polyclonal antibodies (#ab31013, Abcam, Cambridge, UK) (see “Immunohistochemical analysis” in Supplementary Methods). For porcine tooth germ, the paraffin section was stained with Azan staining (see “Azan staining” in Supplementary Methods).

### Extraction and detection of TGF-β and MMP activities in porcine dental pulp

TGF-β and MMPs in porcine dental pulp tissues were extracted and isolated using heparin affinity chromatography. Each fraction was characterized via sodium dodecyl sulfate–polyacrylamide gel electrophoresis (SDS-PAGE), western blotting, zymography and an alkaline phosphatase-human periodontal ligament cell line (ALP-HPDL) system (see “Extraction and detection of TGF-β and MMPs activities in porcine dental pulp” in Supplementary Methods).

### *In vitro* activation of TGF-β1 by MMP2 or MMP11

One microgram of recombinant human latent TGF-β1 (rh-latent TGF-β1, Cell Signaling Technology, Danvers, MA, USA) was dissolved in 50 µL of 50 mM Tris-HCl and 10 mM CaCl_2_ (pH 7.4) and incubated with 1.2 µg of recombinant human MMP2 (rhMMP2) or MMP11 (rhMMP11) (Enzo Life Sciences, Tokyo, Japan) for 20 h at 37 °C. The reaction aliquots at 0 and 20 h were characterized using the ALP-HPDL system (see “Enzyme assay (ALP-HPDL system)” in Supplementary Methods).

### Effect of TGF-β1 on dentin sialophosphoprotein and MMP20 gene expression in dental pulp cells

The cell line PPU-7 was established from porcine dental pulp cells by our group (see “Isolation, transfection and establishment of a porcine dental pulp cell line” in the Supplementary Methods), was plated on a 96-well plate at a density of 1 × 10^4^ cells/well and was cultured in standard medium for 24 h. The growth medium was then changed to a growth medium with or without 1 ng/mL recombinant human transforming growth factor-beta 1 (rh-TGF-β1) (Cell Signaling Technology, Danvers, MA, USA). RNA from PPU-7 cells was extracted at 1, 3 and 7 days using an RNA extraction reagent. The qPCR analysis of total pulp RNA was performed using the SYBR Green technique on a LightCycler Nano system as described in “Quantitative real-time PCR” in the Supplementary Methods. The specific primer sets for full-length DSPP (DSPPv1), DSP only (DSPPv2) and MMP20 and their corresponding reaction conditions are shown in Supplementary Table S1.

### Microcomputed tomography

The dentin mineralization parameters, such as morphology, density, and thickness, in the mandibular incisors of 8-week-old *MMP20* null (KO) and wild-type (WT) mice were analysed via microcomputed tomography (µCT) with a µCT system (μCT-40, Scanco, Bruttisellen, Switzerland). The relative thickness of the dentin layer in cross-sections of mouse mandibular incisors for KO and WT mice was measured using the same system (see “Microcomputed tomography” in the Supplementary Methods).

### Extraction of proteins and detection of TGF-β activity in *MMP20* null mouse dentin

Mouse maxillary and mandibular first molars were extracted from WT (+/+), *MMP20* heterozygous (+/−) and *MMP20* null (−/−) mouse pups using a dissecting microscope at days 5 and 11 (see “Microcomputed tomography” in Supplementary Methods). All the pups were genotyped thereafter using previously reported conditions^51^. The pulp and enamel organ epithelium was removed from the underside of each molar. The mineral was rapidly dissolved by submerging the molars in 2 mL of 0.17 M HCl/0.98% formic acid (HF) for 2 h at 4 °C. Following the removal of undissolved material by centrifugation, the sample was further extracted with 0.5 M acetic acid/2 M NaCl (AN) solution. The AN soluble fraction (AN extract) was dialyzed overnight against water and then lyophilized for analysis via SDS-PAGE, ELISA and the ALP-HPDL system.

### Changes in dentin protein composition and TGF-β activity in dentin

Ten porcine incisors were divided into three regions (R1-R3) at 0.8 cm intervals and ground to a “dentin powder” by means of a jaw crusher (Retsch Inc., Newton, PA, USA). The dentin powder (R1: 2.60 g, R2: 2.37 g, R3: 1.57 g) was sequentially extracted with 50 mM Tris-HCl/4 M guanidine buffer (pH 7.4), 0.17 N HCl and 0.95% formic acid, and 50 mM Tris-HCl/4 M guanidine buffer (pH 7.4) again. The second guanidine extract (G2 extract) was dialyzed against water, lyophilized, and characterized using SDS-PAGE and the ALP-HPDL system (see “Characterization of dentin proteins and TGF-β activity in the enamel formation process in porcine incisors” in the Supplementary Methods).

### Density and volume measurements and protein characterization in porcine root dentin

Each root furcation was prepared from three non-carious porcine first (6^m−1^, 12^m−1^), second (6^m−2^, 12^m−2^) and third (12^m−3^) molars [m = month] (Fig. 7a and b). The parameters for the density measurement of each tooth were determined using a pycnometer with a specific gravity bottle (Wadon), and the density was calculated using the formula described in “Density and volume measurement” in the Supplementary Methods. The volume of each sample was calculated by dividing the weight by the density (see Supplementary Table S4).

Following the density and volume measurement, each root furcation was reduced to tooth powder, and the proteins were extracted using 0.5 M acetic acid/2 M NaCl (AN extract) as described in “Protein extraction after density and volume measurement” in the Supplementary Methods and then characterized by SDS-PAGE and western blotting with 0.3% of the normalized amount (see Supplementary Table S5).

### SDS-PAGE and western blotting

SDS-PAGE was performed using a Novex 4–20% Tris-Glycine gel (Life Technologies/Invitrogen, Carlsbad, CA, USA). The gel was stained with Simply Blue Safe Stain (Invitrogen) or Stains-all Stain (Sigma-Aldrich). The apparent molecular weights of the protein bands were estimated by comparison with the SeeBlue Plus2 Pre-Stained Standard (Life Technologies/Invitrogen). A duplicate of the gel was transblotted onto Invitrolon polyvinylidene difluoride (PVDF) membranes (Life Technologies/Invitrogen) and immunostained with MMP2 (#ab110186, Abcam, Cambridge, UK), MMP11 (#ab53143, Abcam) and porcine DSP (Lampire Biological Laboratories, Pipersvill, PA, USA)^45^ polyclonal antibodies. Immunopositive bands were visualized via enhanced chemiluminescence. Full images of the blots that were cropped in the main figures are shown in Supplementary Fig. S4.

### Zymography

Zymography for MMP was carried out using Novex 10% Zymogram Gelatin Gels (Life Technologies/Invitrogen/Thermo Fisher Scientific, Waltham, MA, USA) (see “Zymography” in the Supplementary Methods).

### Enzyme assay (ALP-HPDL system)

HPDLs were purchased from Lonza (Lonza, Walkersville, MD, USA). The cell culture and ALP activity assays were performed based on the method described in “Enzyme assay (ALP-HPDL system)” in the Supplementary Methods.

### Enzyme-linked immunosorbent assay (ELISA)

Identification of TGF-β1 in HF extracts from the first molars of *MMP20*(+/+), *MMP2*(+/−) and *MMP20*(−/−) mice at days 5 and 11 was achieved with a sandwich enzyme immunoassay method using a Quantikine ELISA kit (R&D Systems, Inc., Minneapolis, MN, USA) (see “Enzyme-linked immunosorbent assay” in the Supplementary Methods).

### Densitometry of DPP and DSP bands on the SDS-PAGE gel

The intensity of the protein bands obtained from the porcine incisors and molars were quantified using ImageJ densitometry software.

### Statistical analysis

For qPCR analysis, enzyme assays using the ALP-HPDL system and measurement of dentin thickness, all values are represented as the mean ± standard error of the mean (s.e.m.). Statistical significance (*) was determined using an unpaired Student’s t-test. In all cases, p < 0.05 was considered statistically significant.

### Data availability

All relevant data are available upon request. Please address requests to Prof. Yasuo Yamakoshi.

## Electronic supplementary material

Supplementary Information

Supplementary Table S1

Supplementary Table S2

Supplementary Table S3

Supplementary Table S4

Supplementary Table S5

Supplementary Fig. S1

Supplementary Fig. S2

Supplementary Fig. S3

Supplementary Fig. S4

Supplementary Fig. S5

Supplementary Fig. S6
