# Supplementary material for: The dynamics of TGF-β in dental pulp, odontoblasts and dentin
Source: Sci Rep. 2018 Mar 13;8:4450. doi: 10.1038/s41598-018-22823-7 (PMC5849713; doi:10.1038/s41598-018-22823-7)
Supplement: Supplementary file 1 — Supplementary Information [file 41598_2018_22823_MOESM1_ESM.pdf]

Supplementary Information

The dynamics of TGF-β in dental pulp, odontoblasts and dentin

Takahiko Niwa[1], Yasuo Yamakoshi[2]*, Hajime Yamazaki[3,4], Takeo Karakida[2], Risako Chiba[2], Jan C-C Hu[5], Takatoshi Nagano[1], Ryuji Yamamoto[2], James P. Simmer[5], Henry C. Margolis[3,4], and Kazuhiro Gomi[1]

[1]Department of Periodontology, School of Dental Medicine, Tsurumi University, 2-1-3 Tsurumi, Tsurumi-ku, Yokohama 230-8501, Japan
E-mail: niwa-takahiko@tsurumi-u.ac.jp; nagano-takatoshi@tsurumi-u.ac.jp; gomi-k@tsurumi-u.ac.jp

[2]Department of Biochemistry and Molecular Biology, School of Dental Medicine, Tsurumi University, 2-1-3 Tsurumi, Tsurumi-ku, Yokohama 230-8501, Japan
E-mail: yamakoshi-y@tsurumi-u.ac.jp; karakida-t@tsurumi-u.ac.jp; chiba-r@tsurumi-u.ac.jp; yamamoto-rj@tsurumi-u.ac.jp

[3]The Forsyth Institute,
245 First Street, Cambridge, MA 02142, USA
E-mail: hyamazaki@forsyth.org; hmargolis@forsyth.org

[4]Department of Developmental Biology, Harvard School of Dental Medicine,
188 Longwood Avenue, Boston, MA 02115, USA

[5]Department of Biologic and Materials Sciences, School of Dentistry, University of Michigan, 1210 Eisenhower Place, Ann Arbor, MI 48108, USA
E-mail: janhu@umich.edu; jsimmer@umich.edu

***Corresponding author:**
Yasuo Yamakoshi, PhD
Professor,
Department of Biochemistry and Molecular Biology
School of Dental Medicine, Tsurumi University
2-1-3 Tsurumi, Tsurumi-ku
Yokohama, 230-8501, JAPAN
Tel.: +81-45-580-8374; Fax: +81-45-573-9599
E-mail: yamakoshi-y@tsurumi-u.ac.jp

Supplementary Note

Extraction of MMPs and TGF-β in porcine dental pulp

Three TGF-β isoforms (TGF-β1 to -β3) have been identified in mammalian. Those isoforms possess over 97% of amino acid sequence homology within mature bioactive molecule[1]. Each of isoforms has also very high amino acid sequence among species (see Supplementary Fig. S2). Certain consensus sequences have been proposed for heparin-binding proteins, such as BBXB or BXBB, where B denotes a positively charged amino acid residue[2]. We found that the mature TGF-β1 and TGF-β2 possess one heparin-binding site conserved in mouse, rat, human and pig (see Supplementary Fig. S2), but not in the mature TGF-β3. Moreover, both MMP2 and MMP11 also possessed its binding site in the catalytic domain (see Supplementary Fig. S3). Based on this finding, we tried to isolate TGF-β isoforms in porcine dental pulp with heparin affinity chromatography.

Immortalization of porcine pulp cells

We established porcine dental pulp-derived cell lines. Following the transfection with pSV3-neo plasmid, we incubated over 20 G418-resistant colonies. However, as most of the cell growth was stopped, we selected 8 clones (PPU-1, -3, -7, -10, -12, -16, -17 and -18).

Because ALP is believed as the initial marker for the differentiation of mesenchymal cells into hard tissue-forming cells such as osteoblasts or odontoblasts[3, 4], we determined the inherent ALP activity of the 8 immortalized pulp cell lines. Both PPU-3 and PPU-7 cell lines possessed the high inherent ALP activity (see Supplementary Fig. S5). For the present study, we selected PPU-7 cell line.

Supplementary Methods

Quantitative real-time PCR

Pulp tip and body, and odontoblasts were extracted with RNA extraction reagent (Isogen, Nippon Gene Co., Ltd., Tokyo, Japan). Following the purified total RNA (2 μg) was reverse transcribed, the reaction mixture consisted of SYBR Green PCR master mix (Roche), supplemented with 0.5 μM forward and reverse primers and 2 μL of cDNA as template. The specific primer sets were designed using Primer-BLAST software (URL: http://www.ncbi.nlm.nih.gov/tools/primer-blast). The specific primer sets and running conditions are shown in Supplementary Table S1 and S2. GAPDH was used as the reference gene. Each ratio was normalized the relative quantification data of TGF-β1, TGF-β2, TGF-β3, latent TGF-β1, latent TGF-β2, latent TGF-β3, TGFBR1, BMP1, two DSPP variants (DSPPv1 and DSPPv2), and MMPs (MMP1, MMP2, MMP3, MMP7, MMP8, MMP9, MMP10, MMP11, MMP13, and MMP20) in comparison to a reference gene (GAPDH) was generated on the basis of a mathematical model for relative quantification in qPCR

system. All values were represented as means ± standard error (s.e.m.). Statistical significance (*) was determined using an unpaired Student's t-test. In all cases, $p<0.05$ was regarded as statistically significant. The resulting data of TGFBR1, latent TGF-β1, latent TGF-β2, latent TGF-β3, TGF-β1, TGF-β2, TGF-β3, TGFBR1, MMP2, MMP20 and MMP11 is shown in main Fig. 1b-k, while that of DSPPv1, DSPPv2 and MMP20 is shown in main Fig. 4c-e.

Immunohistochemical analysis

Mandibles were obtained from postnatal day 11 mice. The mandibles were dissected and fixed with 4% paraformaldehyde for 20 h at 4°C. Hard tissues were decalcified at 4°C in a 10% (w/v) Na_2-EDTA solution (pH 7.0) for 7 days and were embedded in a paraffin[5]. Formalin-fixed paraffin embedded murine mandibles were sectioned with a microtome blade A35 (FEATHER, Osaka, Japan) on a sliding microtome (LS-113, Yamato-Kohki, Asaka, Japan) to produce 4.5 μm-thick sections and were pretreated with 0.3% hydrogen peroxide for 40 min. The sections were treated with 10 mM Tris-EDTA solution (pH 9.0) for 40 min at 80°C as the antigen retrieval procedure, and incubated in a blocking solution (1% BSA, 10% normal goat serum) for 1 h at room temperature. For the primary antibody application, the dilution of anti-TGF-β1 (biorbyt, Cambridege, UK) and anti-TGFBR1 polyclonal antibodies (abcam, Cambridege, UK) was used at 1:500 and 1:300, respectively. For the secondary antibody application, the dilution of HRP-conjugated goat anti-rabbit IgG H&L antibody (abcam, Cambridege, UK) was used at 1:500. The positive signal was detected with 3,3'-diaminobenzidine (DAB) (TaKaRa, Kusatsu, Japan) as a staining substrate. Sections were counterstained to observe clear tissue and cell morphology using hematoxylin. Light micrographs were obtained using a Canon EOS Kiss X8i (Canon, Tokyo, Japan) camera on an optical microscope (OLYMPUS BX50, Olympus, Tokyo, Japan).

Azan staining

Tooth germ was obtained from 6-month-old porcine permanent incisor. The tooth germ was fixed with 4% paraformaldehyde for 20 h at 4°C. Hard tissues were decalcified at 4°C in a K-CX solution (FALMA, Tokyo, Japan) and were embedded in a paraffin. Formalin-fixed paraffin embedded murine mandibles were sectioned with a microtome blade A35 (FEATHER, Osaka, Japan) on a sliding microtome (SM2000R, Leica Biosystems, Nussloch, Germany) to produce 5.0 μm-thick sections. The sections were treated with an equal amount mixture of 10% potassium dichromate (Koso Chemical CO., Ltd. Tokyo, Japan) and 10% trichloroacetic acid (Wako Pure Chemical Industries, Ltd., Osaka, Japan) for 15 min at room temperature. The sections were stained with 0.1% azocarmin G (Merck KGaA, Darmstadt, Germany) in 1% acidic acid solution for 2 h at room temperature. Following the treatment with 0.1% aniline (Wako) and 95% ethanol solution, the reaction was stopped in 0.1% acidic acid and 95% ethanol solution for 1 min. The sections were treated with 5% phosphotungstric acid

(Koso) for 2 h at room temperature and stained with an 0.5% aniline blue-2% orange G mixture. Light micrographs were obtained using Biozero (Keyence, Osaka, Japan).

Extraction and detection of TGF-β and MMPs activities in porcine dental pulp

The frozen dental pulp body (15 g) was minced with a razor blade and suspended with NP40 Cell Lysis Buffer (Life Technologies/Invitrogen, Carlsbad, CA, USA) containing protease inhibitor cocktail (Sigma-Aldrich, St. Louis, MO, USA). The supernatant was buffer-exchanged into 50 mM Tris–HCl and 6 M urea (pH 7.4) with a YM-3 membrane (Merck KGaA, Darmstadt, Germany). The sample was applied onto a Heparin Sepharose 6 Fast Flow column (1.6 cm x 20 cm, GE Healthcare, Uppsala, Sweden) with buffer A: 50 mM Tris–HCl and 6 M urea (pH 7.4). Proteins were eluted with a step gradient of NaCl (0, 0.05, 0.1 and 0.2 M) in buffer A at a flow rate of 0.2 mL min^{-1} at 4°C while monitoring the absorbance at 280 nm. Each fraction was dialyzed against water and characterized by sodium dodecyl sulfate–polyacrylamide gel electrophoresis (SDS-PAGE), Western blot, zymography and an alkaline phosphatase-human periodontal ligament cell-line (ALP-HPDL) system (see "Enzyme assay (ALP-HPDL system)" in the Supplementary Methods.

Isolation, transfection and establishment of cell line of porcine dental pulp cells

Tooth germs of permanent incisors were surgically extracted from the mandibles of deceased 5-month-old pigs (n = 10) from the Meat Market of Metropolitan Central Wholesale Market (Shinagawa, Tokyo, Japan). Pulp tissue pulled out from tooth germs was briefly rinsed in ice-cold sterile PBS to remove blood cells, minced with a surgical blade and digested in a solution of 0.1% collagenase and 0.2% dispase II (Wako Pure Chemical Industries, Osaka, Japan) for 1 hr at 37°C with gentle shaking. The released cells were passed through a 70-µm cell strainer (BD Falcon, Bedford, MA, USA) and washed three times with PBS by centrifugation. The cells were then cultured in alpha Minimum Essential Medium (αMEM) (Gibco/Life Technologies, Carlsbad, CA, USA) containing 10% fetal bovine serum (FBS) and antibiotics (50 U/mL of Penicillin, 50 µg/mL of Streptomycin, Gibco) at 37°C in a humidified 5% CO_2 atmosphere.

Pulp cells isolated from porcine tooth germs were plated at subconfluent cell densities and transfected with the pSV3-neo plasmid (ATCC 37150) using Lipofectamine 2000 (Invitrogen/Life Technologies, Carlsbad, CA, USA) in accordance with the manufacturer's instructions. The pSV3-neo plasmid expresses the SV40 large T-antigen for immortalization and neomycin phosphotransferase for selection. Two days after transfection, the cells were re-plated at low density and selected with 0.35 mg/mL of geneticin (G418) (ATCC, Manassas, VA, USA). The cells were cultured in media containing G418 until colonies were visible. Individual colonies were isolated with cloning cylinders and maintained in the standard medium at 37°C in a humidified

5% CO_2 atmosphere.

Micro-computed tomography

The mice were housed in facilities approved by the Association for Assessment and Accreditation of Laboratory Animal Care. *Mmp20* heterozygous (+/-) mice with C57BL/6 and P129 background were purchased from Mutant Mouse Regional Resource Center (Columbia, MO), and bred to generate wild type (+/+), *Mmp20* heterozygous (+/-) and *Mmp20* null (-/-) mice littermates. All the pups were genotyped at ages at 4-weeks old using the previously reported conditions[6]. Around 8 weeks old, 3 mice from each of WT and KO littermate groups were sacrificed to dissect out their mandibles for micro-computed tomography (μCT) experiments described below. Also, maxillary and mandibular first molars were extracted from mouse pups at postnatal days 5 and 11 of all three genotypes, wild type (+/+), *Mmp20* heterozygous (+/-) and *Mmp20* null (-/-), for protein extraction and detection of TGF-β activity.

Hemi-mandibles of 8-week-old *Mmp20* null (KO) and wild type (WT) mice were dissected out, and fixed with 70% ethyl alcohol. Micro-computed tomography (micro-CT) was used to measure the relative levels of thickness of dentin layer in mouse mandibular incisor cross-sections for KO and WT mice. They were scanned at 8 μm resolution with a micro-CT system (μCT-40, Scanco, Bruttisellen, Switzerland) at 70 kV and 114 mA. The micro-CT images were obtained and analyzed by ImageJ and Amira 3D software. Amira 3D was mainly used to reconstruct 3D images of the hemi-mandibles and to find the right orientation of the incisors in order to accurately measure the dentin mineralization and thickness. The measurement of the dentin mineralization and thickness were performed with an interval of 1 mm from the apex of each incisor to 7 mm distance from the apex towards the incisal tip as previously described[7]. The dentin thickness was measured using the distance tool in Image J software, and the densities of dentin mineralization were determined with the grey scale values of the images in cross-section at 20 μm range under the DEJ and line from DEJ each measurement point using Image J. Density of dentin was estimated based on the gray scale values and internal standards of the micro-CT system.

Characterization of dentin proteins and TGF-β activity in porcine incisor

Following the removal of dental pulp and enamel organ epithelium, as much enamel as possible was removed by scraping with a curette. Ten incisors were cut off by dividing them into three regions (R1-R3) at 0.8 cm intervals and was reduced to “dentin powder” by means of a jaw crusher (Retsch Inc., Newton, PA, USA). Dentin powder (R1: 2.60 g, R2: 2.37 g, R3: 1.57 g) was suspended with 50mM Tris-HCl/4M guanidine buffer (pH 7.4) containing Protease Inhibitor Cocktail Set III [1mM 4-(2-aminoethyl) benzenesulfonyl fluoride hydrochloride (AEBSF), 0.8 mM aprotinin, 50 mM bestatin, 15 mM E-64, 20 mM leupeptin, and 10 mM pepstatin

(Calbiochem EMD Chemicals Inc., Gibbstown, NJ, USA)] and 1 mM 1,10-phenanthroline (Sigma-Aldrich) and was homogenized using a Polytron (Capitol Scientific, Inc., Austin, TX, USA) homogenizer for 30 seconds at half speed. Insoluble material was pelleted by centrifugation (15,900g) and extracted two more times with the same buffer. The guanidine-insoluble material was dialyzed against 16 L of 0.17 N HCl and 0.95% formic acid containing 10mM benzamidine (Sigma-Aldrich), 1 mM PMSF, and 1 mM 1,10-phenanthroline for 1 day. Following centrifugation of the dialysis bag contents, the acid-soluble supernatant was stored at -80°C. The pellet was extracted with 50mM Tris-HCl/4M guanidine buffer (pH 7.4) containing Protease Inhibitor Cocktail Set III and centrifuged, and the supernatant (G2 extract) was dialyzed against water, lyophilized, and characterized by SDS-PAGE and the ALP-HPDL system.

Density and volume measurement

In main Fig. 7, three non-carious porcine first (6m-1, 12m-1), second (6m-2, 12m-2) and third (12m-3) molars [m = month] were used for density and volume measurement (see main Fig.7a). The apical root was first separated by cutting with jewelry saw from 6m-1, 12m-1 and 12m-2 teeth, and then root furcations (approximately 5 mm thick) were prepared by cutting at cementum-enamel junction (CEJ) (see main Fig. 7b). On the other hand, the root furcation from 6m-2 and 12m-3 teeth were obtained by cutting at CEJ with dissecting scissor (see Main Fig. 7b). The

$$m_a = \frac{p_w(T)}{p_w(T')} (m_a' - m_f) + m_f$$

density and volume measurement of root furcation was measured by Pycnometer method. To calculate the mass of pycnometer at T (°C) as the first step, the weight of pycnometer only (m_f) and the weight of pycnometer with water (m_a') were measured. Subsequently, the water density in pycnometer at 25.5°C ($p_w(T)$, before measurement) and at 25.8°C ($p_w(T')$, after measurement) were obtained from the density table of water. The mass of pycnometer (m_a) was determined from the following formula.

As the second step, the weight of root furcation (m_s) and the weight of pycnometer, water and root furcation (m_b) were measured. Subsequently, the water density in pycnometer containing water and root furcation at 25.5°C ($p_w(T'')$) was obtained from the density table of water. The density of root furcation (p_s) was determined from the following formula.

$$p_s\,(g/cm^3) = \frac{m_s}{m_s + (m_a - m_b)} \times p_w(T'')$$

The volume was calculated by dividing weight by density.

$$Vol\,(cm^3) = \frac{m_s}{p_s}$$

The result of each parameter, density and volume is shown in Supplementary Table S4 and the average value of density and volume is shown in main Fig. 7c and 7d, respectively.

Protein extraction after density and volume measurement

Following the density and volume measurement each root furcation was reduced to tooth powder by means of a jaw crusher (Retsch Inc., Newtown, PA, USA). Tooth powder was dialyzed against 10 L of 0.17 N HCl and 0.95% formic acid containing 10mM benzamidine (Sigma-Aldrich), 1 mM PMSF, and 1 mM 1,10-phenanthroline for 3 h at 4°C. Following centrifugation of the dialysis bag contents, the acid-soluble supernatant was stored at -80°C. The insoluble pellet was extracted with 0.5 M acetic acid/2 M NaCl and centrifuged, and the supernatant (AN extract) was dialyzed against 10 L of water for overnight at 4°C, lyophilized, and stored at -80°C. The amount of total protein was normalized by dividing the amount of AN extract by volume (normalized amount), and characterized by sodium dodecyl sulfate–polyacrylamide gel electrophoresis (SDS-PAGE) and Western blotting with 0.3% amount of normalized amount (see Supplementary Table S5).

Zymography

Zymography was carried out using Novex 10% Zymogram Gelatin Gel (Life Technologies/Invitrogen/Thermo Fisher Scientific, Waltham, MA, USA). Samples were dissolved in NuPAGE LDS sample buffer (Invitrogen), and electrophoresis was carried out at 30 mA for about 1 h with Novex Tris Glycine SDS running buffer (Life Technologies/Invitrogen/Thermo Fisher Scientific). The gel was shaken gently in 2.5% Triton X-100 solution for 1 h at room temperature with one buffer change and then incubated overnight with or without 10 mM EDTA in 50 mM Tris–HCl (pH 7.4) containing 10 mM $CaCl_2$. Proteinase activities were visualized as unstained bands after the gel was stained with Coomassie Brilliant Blue R-250 (CBB) (Bio-Rad Laboratories, Hercules, CA, USA). The apparent molecular weights of the protein bands were estimated by comparison with DynaMarker Protein MultiColor III (BioDynamics Laboratory Inc, Tokyo, Japan).

Enzyme assay (ALP-HPDL system)[8]

Human periodontal ligament fibroblasts (HPDL) were purchased from LONZA (LONZA, Walkersville, MD, USA). The HPDL cells were distributed in 96-well plates at a density of approximately 5 x 10^5 cells/well and incubated for 24 hours. The growth medium was changed to contain with or without 10 nM 1α,25-dihydroxyvitamin D_3 and 10 μg mL^{-1} of samples dissolved in ultrapure water. After 72 additional hours of incubation, the cells were washed once with

phosphate buffered saline (PBS), and ALP activity was assayed using 10 mM p-nitrophenylphosphate as the substrate in 100 mM 2-amino-2-methyl-1,3-propanediol-HCl buffer (pH 10.0) containing 5 mM $MgCl_2$ and incubated for 10 minutes at 37°C. Adding NaOH quenched the reaction, and the absorbance at 405 nm was read on a plate reader. Positive controls included the use of recombinant human TGF-β1 (rhTGF-β1) with carrier (0.3 ng mL^{-1}) (R&D Systems). The TGF-β1 receptor inhibitor, SB431542, was applied to a final concentration of 1 mM into the ALP-HPDL system for examination of the influence against the ALP-inducing activity increased by the application of samples. In controls, the ALP-inducing activity in HPDL cells was enhanced by rhTGF-β1.

Enzyme-linked immunosorbent assay (ELISA)

Each of HF extracts (10 µg each) obtained from *Mmp20*(+/+), *Mmp20*(+/-) and *Mmp20*(-/-) mice at days 5 and 11 first molar was bound to TGF-β1 capture antibody coated on the plate and was labeled by HRP-conjugated TGF-β1 detection antibody. The quantitative analysis of TGF-β1 was carried out based upon a calibration curve prepared from different concentrations of standard TGF-β1 (see Supplementary Fig. S6). The positive signal for TGF-β1 was detected using a tetramethylbenzidine (TMB) substrate.

Supplementary References

1. Lyon M, Rushton G, Gallagher JT. The interaction of the transforming growth factor-βs with heparin/heparan sulfate is isoform-specific. *J Biol Chem* **272**, 18000-18006 (1997).

2. Shimazaki K, Uji K, Tazume T, Kumura H, Shimo-Oka T. Approach to identification and comparison of the heparin-interacting sites of lactoferrin using synthetic peptides. *Excerpta Medica Int Congr Ser* **1195**, 37-46 (2000).

3. Sloan AJ, Rutherford RB, Smith AJ. Stimulation of the rat dentine-pulp complex by bone morphogenetic protein-7 in vitro. *Arch Oral Biol* **45**, 173-177 (2000).

4. Chen S, Gu TT, Sreenath T, Kulkarni AB, Karsenty G, MacDougall M. Spatial expression of Cbfa1/Runx2 isoforms in teeth and characterization of binding sites in the DSPP gene. *Connect Tissue Res* **43**, 338-344. (2002).

5. Gao Y, Li D, Han T, Sun Y, Zhang J. TGF-beta1 and TGFBR1 are expressed in ameloblasts and promote MMP20 expression. *Anatomical record (Hoboken, NJ : 2007)* **292**, 885-890 (2009).

6. Caterina JJ, *et al.* Enamelysin (matrix metalloproteinase 20)-deficient mice display an amelogenesis imperfecta phenotype. *J Biol Chem* **277**, 49598-49604 (2002).

7. Smith CE, Hu Y, Richardson AS, Bartlett JD, Hu JC, Simmer JP. Relationships between protein and mineral during enamel development in normal and genetically altered mice. *Eur J Oral Sci* **119 Suppl 1**, 125-135 (2011).

8. Nagano T, *et al.* Porcine enamel protein fractions contain transforming growth factor-β1. *J Periodontol* **77**, 1688-1694 (2006).
